# Case Report: Efficacy of ofatumumab in refractory anti-NMDAR encephalitis: case series and literature review

**DOI:** 10.3389/fimmu.2025.1557210

**Published:** 2025-03-12

**Authors:** Min Deng, Jing Xiong, Dan Hu, Zhaohong Kong, Tao Li

**Affiliations:** Department of Neurology, Renmin Hospital of Wuhan University, Wuhan, China

**Keywords:** NMDAR, encephalitis, ofatumumab, CD20 monoclonal antibody, treatment

## Abstract

Anti-NMDAR encephalitis is the most common autoimmune encephalitis. When first-line treatments fail, second-line therapies are employed. However, a standardized approach for second-line treatment has yet to be established. We presented three cases of anti-NMDAR encephalitis with seizures and psychosis as the primary symptom. These patients showed inadequate response to initial treatments, including intravenous immunoglobulin, methylprednisolone, and plasma exchange. However, their symptoms were effectively controlled following subcutaneous administration of ofatumumab. Previous studies have reported that twelve cases of anti-NMDAR encephalitis were effectively treated with ofatumumab. In this study, the modified Rankin scale (mRS) scores at the last follow-up for all fifteen patients (including our three cases) were significantly lower compared to scores at the peak of the disease (p < 0.001). Thirteen patients achieved full recovery. These findings suggest that CD20 monoclonal antibodies, particularly ofatumumab, may offer a promising treatment option for anti-NMDAR encephalitis.

## Introduction

N-Methyl-D-aspartate receptors (NMDARs) are glutamate-activated ion channels that play a critical role in the central nervous system as the primary excitatory neurotransmitter (1). Anti-NMDAR encephalitis in adults typically presents with psychiatric symptoms, behavioral disturbances, and seizures. The disease predominantly affects female patients (male to female ratio is 1:4) (2). Ovarian teratomas are present in 58% of women aged 18–45 with anti-NMDAR encephalitis (3). Immunopathological studies have demonstrated that the antibody-mediated immune response, rather than cytotoxic T-cell mechanisms, is central to the pathogenesis of anti-NMDAR encephalitis (4). Research further indicates that the B-cell response can lead to an autoimmune reaction against anti-NMDAR that drives encephalitis-like behavioral impairment (5).

First-line immunotherapies commonly used by clinicians include intravenous methylprednisolone (IVMP), intravenous immunoglobulin (IVIG), or plasma exchange (PE). For patients who have not improved within two weeks after receiving two or more first-line therapies, second-line treatments such as rituximab and cyclophosphamide are recommended (6). Rituximab has been shown to be more effective than cyclophosphamide (7), with studies indicating that patients who fail first-line immunotherapy are more likely to achieve independence and experience fewer relapses when treated with rituximab (8). Despite the need for second-line treatments in autoimmune encephalitis, a standardized therapeutic approach remains elusive.

Ofatumumab is a novel fully human monoclonal antibody that selectively eliminates B lymphocytes and has a low immunogenic risk profile. Ofatumumab exhibited enhanced *in vitro* complement-dependent cytotoxicity activity compared with rituximab (9). Is ofatumumab suitable for the second-line treatment of anti-NMDAR encephalitis? In the current case report, one patient experienced rapid relapse and two patients showed poor response to first-line treatment. All three patients subsequently received ofatumumab and achieved favorable outcomes.

## Case 1

### Case presentation

A 47-year-old female patient began to experience psychiatric symptoms and behavioral disorders in August 2022. The main symptoms include insomnia, walking back and forth, agitation, unable to communicate, and talking to himself. She was hospitalized in the psychiatric department in August 2022. She was diagnosed with mood disorder. During her hospitalization, the patient showed tonic-clonic seizures and was transferred to the department of neurology. Video electroencephalogram(EEG)showed generalized slow wave (delta wave) discharges during sleep. Analysis of her cerebrospinal fluid (CSF) showed normal opening pressure (165 mm H_2_O), white blood cells (1×10^6^), protein (0.28g/L), and glucose levels (2.75mmol/L). Complete blood count indicated elevated white blood cells and neutrophil, with decreased monocytes and eosinophil. Elevated procalcitonin (PCT) level was detected. MRI scans of the head and pelvis were normal. Anti-NMDAR IgG was detected positive in her serum (1:100) and cerebrospinal fluid (1:10). She was diagnosed with anti-NMDAR encephalitis. She was treated with sodium valproate, levetiracetam and IVIG (0.4 mg/kg for 5 days). Her symptoms persisted despite IVMP treatment (1000 mg/day, tapered every 3 days) and four sessions of PE. In addition, she also received antibacterial treatment and was discharged from the hospital in October 2022.

### Outcome and follow-up

In March 2023, a follow-up brain MRI showed no abnormalities, but video-EEG revealed significant slow-wave activity during wakefulness and sleep. The anti-NMDAR IgG was detected positive in her serum (1:10) and cerebrospinal fluid (1:3.2). She was readmitted to the hospital due to irritability and talking to herself in June 2023. The EEG showed generalized slow wave (delta wave) discharges during wakefulness and sleep. In addition, she suffered from seizures for a long time. The anti-NMDAR IgG was detected positive in her serum (1:100). Given her rapid relapse, she received three injections of ofatumumab (20mg subcutaneous). No drug allergies or adverse reactions occurred. The EEG normalized five weeks post-treatment.

**Figure 1:**
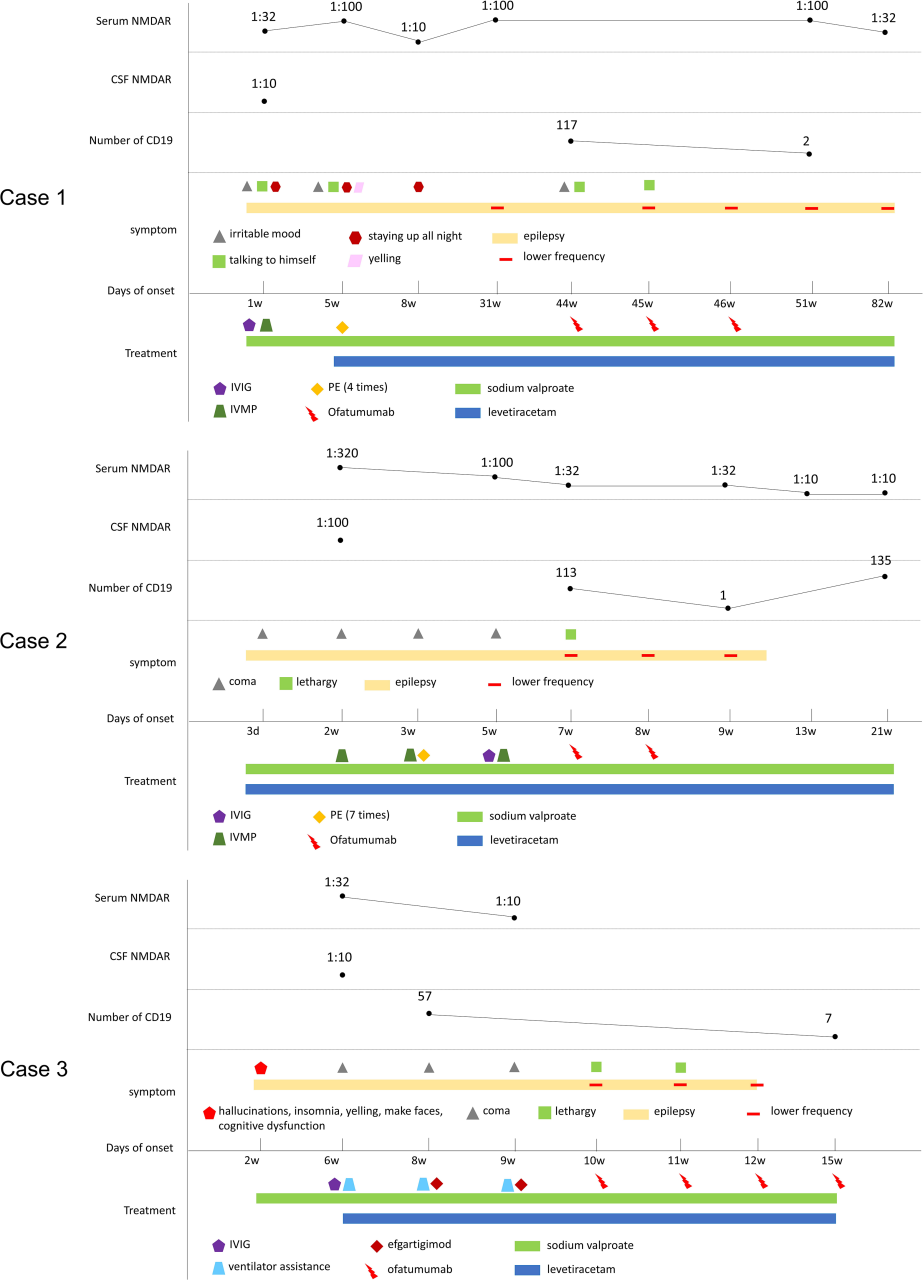
Schematic illustration of the disease course and follow-up of three cases. CSF, cerebrospinal fluid; anti-NMDAR, anti-N-methyl-D-aspartate receptor antibody; IVMP, intravenous methylprednisolone; IVIG, intravenous immunoglobulins; PE, plasma exchange.

The patient received a total of 3 injections of ofatumumab. The number of CD19 dropped from 117 to 2. Now the patient’s seizures symptoms were controlled, and her symptoms of irritability and talking to herself have improved significantly. No adverse drug reactions were observed. The clinical manifestations, diagnosis, treatment methods and follow-up of the patients are detailed in **Figure 1**.

## Case 2

### Case presentation

A 20-year-old male patient began to experience epileptic seizures in September 2023. Each episode lasted approximately 2 minutes and occurred 2-3 times daily. During the seizures, the patient was unresponsive. He was hospitalized in September 2023. The video EEG showed continuous spikes and waves during sleep. Analysis of the cerebrospinal fluid showed normal opening pressure (160 mm H2O), elevated white blood cells (13×10^6^), normal protein (0.36 g/L) and glucose levels (3.42 mmol/L). The CT scan of the chest showed infection in the lower lobe of the left lung. The blood cell count showed that the elevated leukocyte and neutrophil, and the decreased monocyte. Elevated PCT levels were detected. The MRI scans of the brain were unremarkable. Anti-NMDAR IgG was positive in serum (titer 1:320) and cerebrospinal fluid (titer 1:100). He was diagnosed with anti-NMDAR encephalitis. He was treated with sodium valproate extended-release tablets and levetiracetam. In addition, he also received IVMP (1000 mg/d, reduced by half every 3 days). Although seizure frequency decreased, the patient remained unconscious. The patient received seven PE sessions and a 5-day course of intravenous immunoglobulin (IVIG) therapy (0.4 g/kg/day). By November 2023, his consciousness improved to a stuporous state. Given the inadequate response to first-line treatment, he received two ofatumumab injections. No drug allergies or adverse reactions occurred.

### Outcome and follow-up

The patient received two injections of ofatumumab (20mg subcutaneous). The number of CD19 dropped from 113 to 1. The patient’s seizures symptoms are now well controlled. No adverse drug reactions occurred. The clinical manifestations, diagnosis, treatment methods and follow-up of the patients are detailed in **Figure 1**.

## Case 3

### Case presentation

A 32-year-old male patient had experienced hallucinations, insomnia, yelling, facial grimacing, and cognitive dysfunction starting in February 2024. Initial evaluation at a community hospital included a normal brain MRI. His EEG examination showed generalized theta waves discharges during sleep, especially in the frontal lobe. He was treated with lipidone oral solution (1ml BID) and sodium valproate (500mg BID). After 1.5 months of treatment, his above symptoms gradually worsened, and he developed respiratory distress, necessitating transfer to our hospital on March 27, 2024. He immediately received the tracheal intubation and ventilator auxiliary respiratory treatment. The CSF analysis shows opening pressure (160 mm H2O), elevated white blood cells (11×10^6^), normal protein (0.35g/L) and glucose levels (3.42 mmol/L). The blood cell count showed that the elevated white blood cells. Elevated alanine aminotransferase levels were detected. CT scanning of the lungs shows the inflammation of the lower lobe in both sides. Anti-NMDAR IgG was detected positive in her serum (1:32) and cerebrospinal fluid (1:10). He was diagnosed with anti-NMDAR encephalitis. He was treated with sodium valproate and levetiracetam. When he also received 5-day injections of IVIG (0.4g/kg/d) and two injections of efgartigimod, patient regained consciousness but remained lethargic and breathed autonomously without the help of the ventilator. In addition, his seizures were controlled. Then he received four subcutaneous injections of ofatumumab (20 mg each) on April 23, April 30, May 6 and May 27. By the second injection, his consciousness returned to normal. No drug allergies and adverse reactions occurred.

### Outcome and follow-up

**Figure 2:**
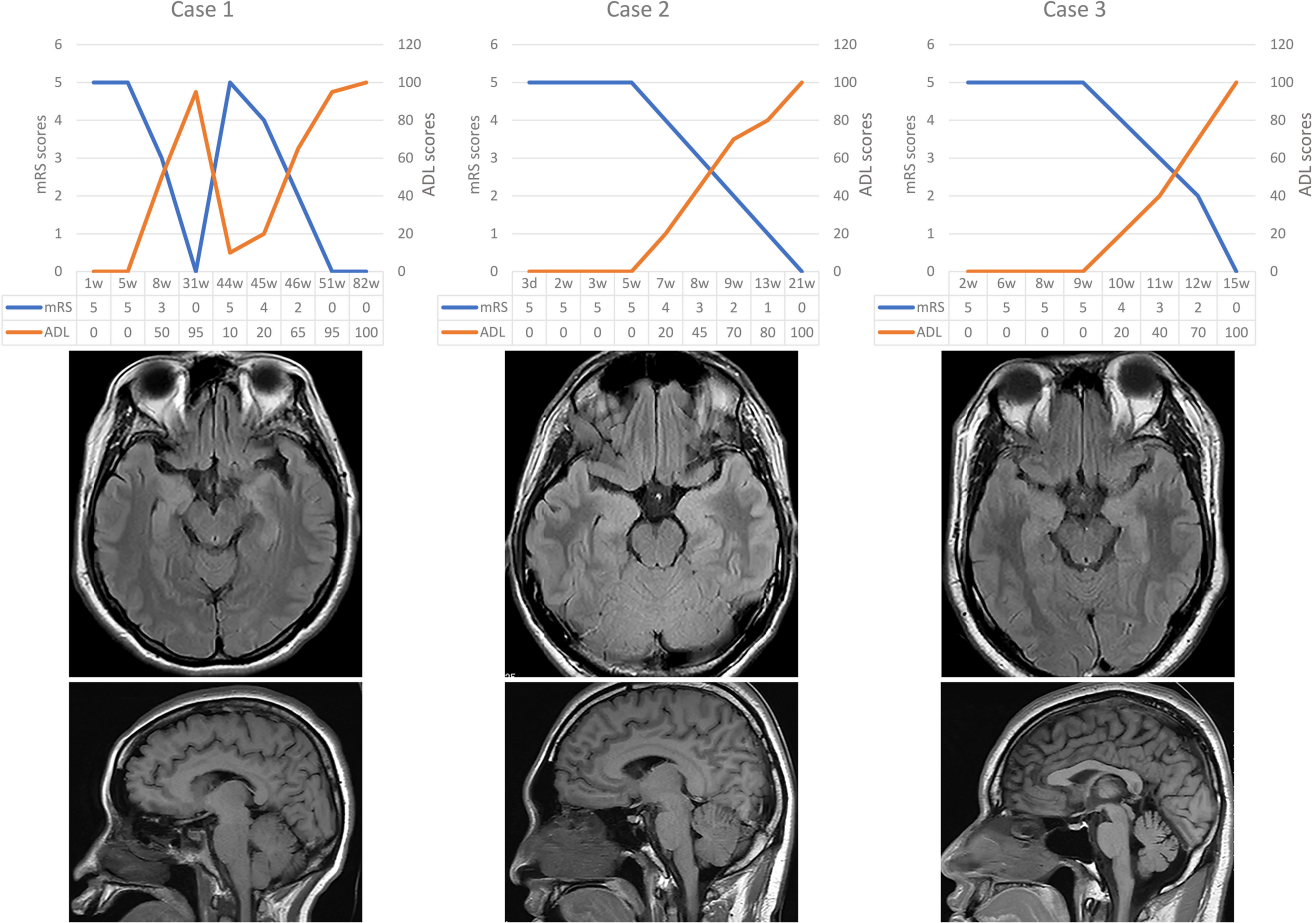
Brain imaging and the scores of mRS and ADL for three cases. mRS, modified Rankin scale; ADL, activities of daily living.

He received a total of 4 injections of ofatumumab (20mg once, subcutaneous). The number of CD19s dropped from 57 to 7. All symptoms resolved without adverse drug reactions. The Patient is currently treated with sodium valproate and levetiracetam (**Figure 1**). The modified Rankin scale (mRS), the Barthel Index for activities of daily living (ADL) and cerebral MRI scan of the three patients mentioned above are showed in **Figure 2**.

## Literature search

The literature search strategy followed the guidelines provided by the Preferred Reporting Items for Systematic Reviews and Meta-Analyses. The terms “NMDAR” and “ofatumumab” were used to search in the PubMed database, and the data were retrieved until December 30, 2024. To provide a comprehensive overview of patient characteristics, patients with incomplete demographic and serological findings and undetailed treatment information were excluded. The following cases were excluded: cases without encephalitis events; cases with positive antibodies in addition to anti-NMDAR antibodies; cases without ofatumumab treatment in the acute phase; Lack of records on acute treatment and follow-up information.

**Figure 3:**
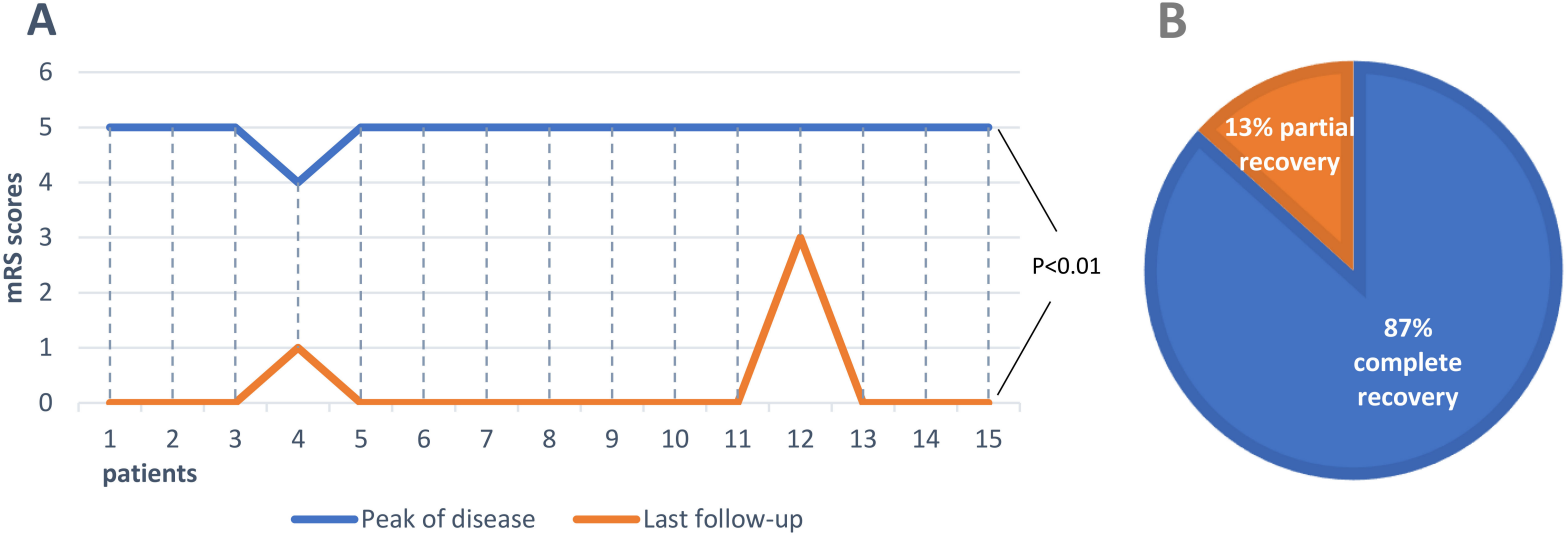
Clinical outcomes in all anti-NMDAR encephalitis patients treated with ofatumumab. **(A)** Comparison of mRS scores at peak of the disease with that at the last follow-up in 15 patients. **(B)** Proportion of outcomes in 15 patients.

Five articles were screened in PubMed (10–14). Information of the enrolled patients is shown in **Table 1**. Fifteen patients, including the three patients in this study, were selected for further review. Five of the fifteen patients were male (33%). Ages of onset ranged from 11 to 75 years, with a median of 18 years. The main clinical manifestations were psychotic behavior (87%), epilepsy and cognitive impairment (80%), behavioral disorders (53%), insomnia and vegetative symptoms (33%), autonomic dysfunction (27%), and involuntary movement (13%). All cases had a head MRI scan before the start of acute immunotherapy. This MRI scan showed abnormalities in the cerebral cortex (33%) and hippocampal heads and medial temporal lobe (13%). Except for one case with teratoma, the remaining cases had no signs of tumor. The CSF anti-NMDAR antibodies were confirmed in all fifteen cases, and thirteen cases are at titers in the range of 1:10 to 1:100. Serum anti-NMDAR antibody testing of five patient was negative. Serum anti-NMDAR antibody testing of the other ten patients was positive, with titers ranging from 1:10 to 1:1000. The most common first-line therapy was IVMP, which was used in thirteen patients. After receiving one or more immunotherapy (IVMP, IVIG, MMF, PE), these cases still could not be effectively treated and selected ofatumumab subcutaneously. 67% cases took hormones or seizures drugs for a long time. The average mRS score (0.27) at last visit for the 15 patients was significantly lower compared with the average mRS score (4.9) at the peak of the disease (**Figure 3A**, p<0.001). 87% cases have achieved full recovery (**Figure 3B**).

**Table 1:** Overview of cases included in this study.

Case	Age/sex	Symptoms	Other disease or family history	MRI lesions	Tumor	NMDAR-ab		Doses of Ofatumumab	Other acute immunotherapy	Long-term immunotherapy	Outcome
						CSF	Serum				
1 (10)	20+/F	Seizure, agitation, hallucination, consciousness disturbance	No	Normal	No	1:32	1:320	3	IVMP, IVIG, MMF	No	Full recovery
2 (10)	30+/M	Behavioral changes, hallucinations, speech disturbances, and confusion, insomnia and vegetative symptoms	No	Cerebral cortex	No	1:10	normal	1	IVMP	No	Full recovery
3 (11)	18/F	Psychosis, behavioral disorders, irritability, insomnia	Not available	Not available	No	1:100	1:30	4	IVMP, IVIG	No	Full recovery
4 (12)	75/F	Impulsiveness, irritability, aggressive behavior,	Not available	Not available	No	1:1	1:10	2	IVIG	No	Partial recovery
5 (13)	18/F	Seizure, psychotic behavior	No	bilateral hippocampal heads, left medial temporal lobe	No	1:10	1:10	1	IVMP, MMF,	Oral glucocorticoids	Full recovery
6 (13)	15/F	Seizures, consciousness disturbance, psychiatric symptoms, autonomic dysfunction, involuntary movement	No	left cerebral hemisphere	No	1:10	1:32	2	IVMP, IVIG,	Oral glucocorticoids	Full recovery
7 (13)	16/M	Seizures, consciousness disturbance, psychiatric symptoms, autonomic dysfunction, involuntary movement	No	bilateral cerebral hemispheres parenchyma, meninges	No	1:30	1:10	2	IVMP, IVIG, Efgartigimod	Oral glucocorticoid	Full recovery
8 (14)	14/F	Seizure, consciousness disturbance, memory deficit, agitation, behavioural changes	No	bilateral frontal subcortical white matter	No	1:1	Negative	3	IVMP; IVIG	No	Full recovery
9 (14)	12/M	Seizure, consciousness disturbance, memory deficit, agitation, behavioral changes	No	Normal	No	1:10	Negative	4	IVMP; IVIG	Oral glucocorticoid	Full recovery
10 (14)	11/F	Seizure, consciousness disturbance, severe insomnia, agitation, behavioural changes, a decrease of verbal output	No	bilateral periventricular white matter	No	1:10	Negative	3	IVMP; IVIG	Oral glucocorticoid	Full recovery
11 (14)	11/F	Seizure, consciousness disturbance, severe insomnia, agitation, behavioural changes, a decrease of verbal output, dyskinesias, dystonia	No	bilateral frontal lobe, temporal lobe and parietal lobe	No	1:30	Negative	4	IVMP; IVIG; IA	Oral glucocorticoid	Full recovery
12 (14)	11/F	Behavioural changes, consciousness disturbance, seizure, agitation, a decrease of verbal output, dyskinesias, dystonia	Pneumonia, teratoma	bilateral globus pallidus, caudate nucleus, frontal lobe, temporal lobe and insular cortex	Teratoma	1:320	1:1000	5	IVMP; IVIG; PE; IA	Oral glucocorticoid	Partial recovery

M, male; F, female; NA, not available; NMDAR-ab, anti-N-methyl-D-aspartate receptor antibody; IVMP, intravenous methylprednisolone; IVIG, intravenous immunoglobulins; MMF, mycophenolate mofetil; PE, plasma exchange.

## Discussion

All the cases, including three cases in our study, experienced short-term relapse or poor treatment response following IVMP, IVIG MMF or PE treatments. Their symptoms were improved after receiving ofatumumab injections, with no drug-related adverse reactions observed.

Ofatumumab effectively mediates B cell lysis through complement-dependent cytotoxicity and antibody-dependent cytotoxicity (15). Ofatumumab was approved by the U.S. Food and Drug Administration in 2020 for the treatment of relapsing forms of multiple sclerosis in the field of neuroscience. The adverse events include respiratory tract infection and immunogenicity. The administration method of ofatumumab is simple and can be administered by subcutaneous injection at home. Previous case reports have shown that its efficacy in adult anti-NMDAR encephalitis (10, 12), and in three patients with MOG and anti-NMDAR IgG double-positive encephalitis (11, 16). In this study, all three patients showed clinical improvement post-ofatumumab treatment, with efficient depletion of CD19+ B cells.

The first-line treatment improved symptoms in 53% of anti-NMDAR encephalitis patients (17), with 75% achieving recovery or mild sequelae. However, 25% of patients with anti-NMDAR encephalitis may suffer severe neurological damage or even death (18), and recurrence rates range from 12% to 24% (19). However, ofatumumab treatment resulted in complete recovery in 87% of patients with refractory or relapsed anti-NMDAR encephalitis in this review. For refractory anti-NMDA antibody encephalitis or short-term relapse, second-line treatment should be initiated as soon as possible. Common second-line treatments include cyclophosphamide and rituximab targeting the depletion of B lymphocytes. The B lymphocytes play an important role in the pathogenesis of the anti-NMDAR autoimmune encephalitis.

Cyclophosphamide has good bioavailability within the central nervous system. However, due to its potential for serious side effects, such as bone marrow suppression, infertility, hemorrhagic cystitis, and increased risk of malignancy, it is generally less preferable than rituximab as a second-line agent for anti-NMDAR encephalitis (20). Compared with rituximab and ocrelizumab, ofatumumab is a fully human anti-CD20 monoclonal antibody. Patients do not need to receive the treatment of prophylactic antihistamines and acetaminophen prior to dosing. In addition, compared with rituximab and ocrelizumab only binding to one epitope of CD20, ofatumumab binds to different conformational epitopes (21). Lnebilizumab is directed against the CD19 B cell surface antigen. Compared with rituximab, inebilizumab not only depletes CD20+ B cells but also eliminates plasma cells, resulting in more extensive suppression of B cells (22). Daratumumab is an anti-CD38 monoclonal therapeutic antibody approved for the treatment of refractory multiple myeloma. Of note, daratumumab had minimal effect on serum anti-NMDAR titers, which raised questions about the role of this drug in AE (22). In this review, ofatumumab demonstrated excellent outcomes with no adverse effects.

## Conclusion

Satisfactory clinical outcomes were observed after the use of ofatumumab that promoted B cell depletion in the treatment of anti-NMDAR encephalitis in these cases. Notably, ofatumumab could be considered as an alternative to rituximab and cyclophosphamide for the treatment of anti-NMDAR autoimmune encephalitis. However, further studies are needed to validate its efficacy.

## Data Availability

The original contributions presented in the study are included in the article/supplementary material. Further inquiries can be directed to the corresponding author.
